# Cytomegalovirus shedding in seropositive healthy women of reproductive age in Tianjin, China

**DOI:** 10.1017/S0950268820000217

**Published:** 2020-02-19

**Authors:** D. Ju, X. Z. Li, Y. F. Shi, Y. Li, L. Q. Guo, Y. Zhang

**Affiliations:** 1 Department of Gynaecology & Obstetrics, Prenatal Diagnostic Centre, Tianjin Medical University General Hospital, Tianjin, China; 2 Department of Occupational & Environmental Health, School of Public Health, Tianjin Medical University, Tianjin, China

**Keywords:** Cytomegalovirus, reproductive age, seropositive women, shedding

## Abstract

Cytomegalovirus (CMV) enters latency after primary infection and can reactivate periodically with virus excreted in body fluids which can be called shedding. CMV shedding during the early stage of pregnancy is associated with adverse pregnancy outcome. The shedding pattern in healthy seropositive women who plan to have babies has not been well characterised. Vaginal swabs, urine and blood were collected from 1262 CMV IgG-positive women who intended to have babies and tested for CMV DNA by fluorogenic quantitative PCR method. Serum IgM was also detected. The association between sociodemographic characteristics and CMV shedding prevalence was analysed. Among 1262 seropositive women, 12.8% (161/1262) were detected CMV DNA positive in at least one body fluid. CMV DNA was more frequently detected in vaginal secretion (10.5%) than in urine (3.2%) and blood (0.6%) also with higher viral loads (*P* < 0.00). CMV shedding was more likely detected in IgM-positive women than IgM-negative women (29.5% (13/44) *vs.* 12.2% (148/1218); OR 3.03, 95% CI 1.55–5.93; *P* = 0.001). CMV shedding in vaginal secretion was highly correlated with shedding in urine, the immune state of IgM, the adverse pregnant history and younger age. CMV shedding was more commonly detected in vaginal secretion than in urine or blood with higher viral loads among healthy seropositive women of reproductive age. Further studies are needed to figure out whether the shedding is occasional or continuous and whether it is associated with adverse pregnancy outcomes.

## Introduction

Cytomegalovirus (CMV) infection is one of the most common causes of intrauterine infection and congenital infectious disease, which can result in sensorineural hearing loss and intellectual disability [1, 2]. CMV infection is ubiquitous in the general population and does rarely cause symptomatic disease in immunocompetent individuals but can cause serious clinical consequences in special population like immunocompromised individuals and foetus. It is documented that the seroprevalence of CMV in pregnant women in developed countries is about 40–50% [3], but over 90% in developing countries such as China [4–6]. In contrast to other congenital infections like rubella and toxoplasmosis, preconception maternal immunity of CMV provides only partial protection to prevent the transmission to the foetus [7, 8]. Likewise, plenty of evidence show that congenital CMV infection happens following maternal reactivated infection or reinfection as well as primary infection [9–12]. Moreover, in the high seroprevalence country, a large proportion of congenital CMV infection happens following maternal non-primary infection instead of primary infection [9, 10]. The virus becomes latent in the host after primary infection and the host sheds CMV intermittently in body fluids such as urine, saliva and other body secretions as a result of either primary or non-primary infection (including reactivated infection and reinfection) [13]. Previous studies have disclosed that CMV shedding during gestation is associated with adverse pregnancy outcomes especially in the first trimester of pregnancy [14, 15]. Therefore, understanding the shedding pattern of women who plan to have babies is of great importance.

CMV shedding pattern was well studied in pregnant women, children, postpartum women and immunocompromised populations [16–20]. However, the available data about the CMV shedding mode in immunocompetent seropositive non-pregnant women of reproductive age are limited and the sample size in the previous studies is small. It is meaningful to study the CMV shedding pattern in seropositive women of child-bearing age including the occurrence of CMV shedding in different body fluids, the virus loads and the risk factors associated with CMV shedding. It can contribute to a better understanding of the CMV shedding prevalence in women of child-bearing age and provide data for the scheme of precaution and control of CMV infection in such population.

## Methods

### Study design and population

The objectives of the study are to understand the CMV shedding status in different body fluids among seropositive non-pregnant women of child-bearing age and the correlation between CMV shedding and related risk factors.

The study was approved by the Ethical Committee of Tianjin Medical University General Hospital (Ethical NO. IRB2019-WZ-083). Informed consent was obtained from each participant when enrolling.

From October 2013 to July 2018, women of child-bearing age in the healthy state who came to the Prenatal Diagnostic Centre in the General Hospital of Tianjin Medical University for pre-pregnancy checks were tested for CMV IgG and CMV IgM status first. The history of menstruation was collected; those who were suspected to be pregnant were excluded from the study. Women with HIV-positive and malignant tumour were also excluded. IgG-positive non-pregnant women with IgM either positive or negative were recruited when they agreed to participate. Blood, urine and vaginal swabs were obtained simultaneously to test CMV DNA within 1 week after the test of serum. A standard questionnaire was administrated to collect the sociodemographic characteristics and the basic information of the participants, including the age, residence, education background, occupation, gravidity, parity history and adverse pregnancy history.

### Sample collection and laboratory assay

Blood samples were collected in vacuum blood collection tubes with coagulant or anticoagulant (EDTA-K2), respectively, for CMV antibody test and DNA extraction. Mid-stream urine samples were collected in sterile tubes. Vaginal swabs were collected following a standard operating procedure. A sterile cotton swab is placed at the posterior vaginal fornix rotated for 15–30 s. Swabs with secretion were immersed directly in collection tubes filled with 1 ml saline as the transport buffer and were soaked for at least 60 min before the test. Eligible samples were sent to the laboratory and tested within 72 h.

CMV IgG antibody in serum was tested by enzyme-linked immunosorbent assay kit (Bell Biological Technology, Beijing, China) following the recommended instructions. Seropositive women were detected for CMV DNA in blood, urine and vaginal secretion. Total DNA in blood (peripheral blood mononuclear cells), urine and vaginal secretion was extracted using TIANamp Genomic DNA kit (TIANGEN BIOTECH company Beijing LTD, Beijing, China). The presence of CMV DNA was detected using a commercial diagnostic kit (DaAn Gene Company, Guangzhou, China) by fluorogenic quantitative PCR following the manufacturer's protocol. A highly conserved non-coding region in IE1 gene encoding the immediate early transcription regulation proteins of HCMV-AD169 strain was amplified as a target region. The amplified fragment length was 86 bp. Five microliters of the extracted DNA sample was added to CMV-PCR reaction tube with the reaction mix for amplification as previously described [21]. Amplification was performed with an ABI Prism 7500 Real Time Cycler (Applied Biosystems, Foster City, CA, USA) under the thermal profile of 93 °C for 2 min, 10 cycles of 93 °C for 45 s and 55 °C for 60 s, followed by 30 cycles of 93 °C for 30 s and 55 °C for 45 s. When the cycle thresholds of PCR were <30 times, the virus copies were >1 × 10^3^ copies/ml and were diagnosed with CMV DNA positive. The limit of detection of this assay is 1000 copies/ml (3.00 log10 copies/ml) according to the standard curve calibrated by reference standards.

### Data analysis

Statistical analysis was performed by SPSS 19.0 software. Women were defined as positive with CMV shedding by detecting CMV DNA in at least one body fluid (blood, urine or vaginal secretion). Continuous variables were described by mean and standard deviation (s.d.) when normally distributed. Otherwise, they were described by medians and ranges. *P*-value was calculated by using *χ*^2^, continuity correction or Fisher's exact tests as appropriate. Adjusted *P*-values for pairwise multiple comparisons were calculated by Bonferroni correction. Kruskal–Wallis test was used to compare the virus copies among the three different body fluids groups. Mann–Whitney *U* test was used to compare the difference when the parameters were non-normally distributed. The univariate and multivariable logistic models were adopted to examine the associations between the sociodemographic characteristics and CMV shedding in vaginal secretion.

## Result

A total of 1262 CMV IgG-positive women of reproductive age were enrolled in the study，including 1218 (96.5%) who were IgM-negative and 44 (3.5%) who were IgM-positive. All the participants were not pregnant. The median age was 30 years old (range from 20 to 45). Most (80.6%) of the women were between 25 and 34 years old. Sixty-eight (5.4%) were <24 years old and 177 (14%) were over 35 years old. A majority of women (86.8%) came from urban areas. One hundred and seventy (13.5%) of them had <9-year basic education (Chinese 9 years compulsory education from primary school to junior high school). Sixty (4.8%) of them had a Master's degree or higher. A total of 908 (71.9%) engaged in non-manual work while 130 (10.3%) engaged in manual work. A total of 379 (30%) of them had a history of adverse pregnancy outcomes just like spontaneous abortion, stillbirth, intrauterine foetal death, congenital birth defects and intrauterine growth retardation. A total of 282 (22.3%) were multipara. The basic characteristics of the study subjects were shown in Table 1.

**Table 1.** Basic characteristic of the study population

Characteristics	Total no. *n* = 1262 (%)
Age	
⩽24 years	68 (5.4)
25–34 years	1017 (80.6)
⩾35 years	177 (14.0)
Region	
Urban	1096 (86.8)
Rural	166 (14.5)
Education background	
Less than high school	170 (13.5)
Junior college	402 (31.9)
Bachelor's degree	630 (49.9)
Master's degree or higher	60 (4.8)
Occupation	
Unemployment	224 (17.7)
Manual work	130 (10.3)
Non-manual work	908 (71.9)
Adverse pregnancy history	
No	883 (70.0)
Yes	379 (30.0)
Parity history	
None	980 (77.7)
⩾1	282 (22.3)
IgM	
Seronegative	1218 (96.5)
Seropositive	44 (3.5)
CMV shedding in at least one body fluid	
No	1101 (87.2)
Yes	161 (12.8)

**Fig. 1.**
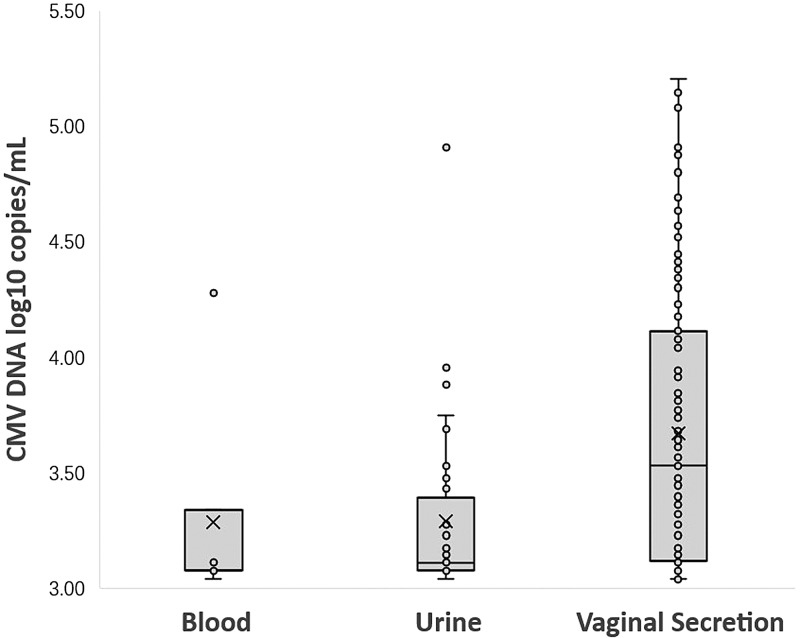
CMV DNA loads (log10 copies/ml) in blood, urine and vaginal secretion samples with positive PCR results. (Samples with CMV loads <3.00 log10 copies/ml were considered negative.)

Among 1262 seropositive women, 12.8% (161/1262) were detected CMV DNA-positive in at least one body fluid. A total of 1.4% (18/1262) women shed CMV in two different body fluids simultaneously. None had CMV shedding in three body fluids at the same time. A total of 0.6% (7/1262) women were detected CMV DNA-positive in blood, the median CMV viral load in blood was 3.08 log_10_ copies/ml (range from 3.04 to 4.28 log_10_ copies/ml); CMV DNA was detected in 3.2% (40/1262) of the urine specimens, the median CMV viral load in urine was 3.11 log_10_ copies/ml (range from 3.04 to 4.91 log_10_ copies/ml), only one (2.5%, 1/40) woman was detected with virus loads exceeded 4.0 log_10_ copies/ml. A total of 10.5% (132/1262) women were detected CMV DNA-positive in vaginal secretion. The median CMV viral load in vaginal secretion was 3.53 log_10_ copies/ml (range from 3.04 to 5.20 log_10_ copies/ml), of which, 29.5% (39/132) women were detected with higher virus loads (＞4.0 log_10_ copies/ml). The prevalence of CMV shedding was statistically different in the three body fluids (*P* < 0.000). CMV DNA was more frequently detected in vaginal secretion than in urine and blood and the viral loads were significantly higher in vaginal secretion (*P* < 0.000) which was shown in Table 2 and Figure 1.

**Table 2.** CMV shedding prevalence and the viral loads in different body fluids

Body fluids	CMV shedding detected			CMV DNA copy number	
	*n* (%) *N* = 1262	95% CI	*P-value	Median: log_10_ copies/ml (range)	**P-value
Blood	7 (0.6)	0.1–1.0		3.08 (3.04–4.28)	
Urine	40 (3.2)	2.2–4.1		3.11 (3.04–4.91)	
Vaginal secretion	132 (10.5)	8.8–12.2	0.000	3.53 (3.04–5.20)	0.000

Samples with CMV copies >1 × 10^3^ copies/ml (3.00 log10 copies/ml) were diagnosed positive and considered as samples with CMV shedding detected. Samples with CMV loads <3.00 log10 copies/ml were considered negative.

*The CMV prevalence is statistically different among the three body fluids and the difference of CMV prevalence between each two body fluids is statistically significant by pairwise comparison with adjusted *P*-value.

**The viral loads among the three different body fluids are statistically different by Kruskal–Wallis test. The difference of CMV DNA copy number between urine and vaginal secretion was statistically significant (adjusted *P* = 0.000). There was no significant difference between the CMV DNA copy number in blood and urine (adjusted *P* = 1.000), and there was also no significant difference between blood and vaginal secretions (adjusted *P* = 0.059).

According to the multivariant analysis, CMV shedding in vaginal secretion was strongly associated with the presence of CMV DNA in urine, the presence of serum IgM and the adverse pregnancy history. Younger age was also a risk factor. The prevalence of CMV shedding in vaginal secretion decreased with age (17.6%, 10.2%, 9.0% in those who aged 20–24, 25–34, 35–45 years, respectively). In contrast to women aged 20–24, those aged 35–45 were less frequently detected with virus shedding in vaginal secretion (*P* = 0.040). Shedding in vaginal secretion and urine was highly correlated (OR 5.66, 95% CI 2.85–11.24). In addition, women with adverse pregnancy history had a higher shedding prevalence in vaginal secretion (OR 1.53, 95% CI 1.05–2.25), which were shown in Table 3.

**Table 3.** Analysis of the relationship between CMV shedding in vaginal secretion and relevant factors

Variables	Total	CMV shedding in VS	No CMV shedding in VS	Univariant		Multivariant	
	*N* = 1262	*n* = 132 *n* (*N*%)	*n* = 1130 *n* (*N*%)	*P*-value		OR (95% CI)	Adjusted *P*-value
Age					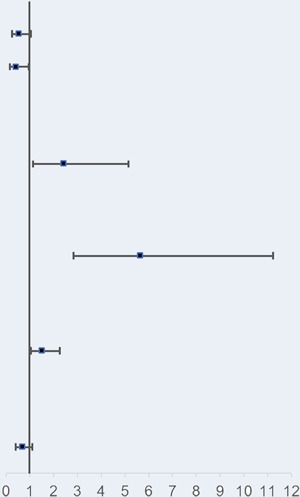		
⩽24 years	68	12 (17.6)	56 (82.4)			ref	
25–34 years	1017	104 (10.2)	913 (89.8)			0.53 (0.27–1.04)	0.066
⩾35 years	177	16 (9.0)	161 (91.0)	0.123		0.42 (0.18–0.96)	0.040
IgM							
Seronegative	1218	121 (9.9)	1097 (90.1)			ref	
Seropositive	44	11 (25.0)	33 (75.0)	0.003*		2.42 (1.14–5.15)	0.022
Shedding in urine							
No	1222	116 (9.5)	1106 (90.5)			ref	
Yes	40	16 (40.0)	24 (60.0)	0.000*		5.66 (2.85–11.24)	0.000
Adverse pregnancy history							
No	883	79 (8.9)	804 (91.1)			ref	
Yes	379	53 (14.0)	326 (86.0)	0.007		1.53 (1.05–2.25)	0.029
Parity history							
None	980	110 (11.2)	870 (88.8)			ref	
⩾1	282	22 (7.8)	260 (92.2)	0.098		0.69 (0.42–1.12)	0.133
Shedding in blood							
No	1255	131 (10.4)	1124 (89.6)				
Yes	7	1 (14.3)	6 (85.7)	1.000*			
Region							
Urban	1096	110 (10.0)	986 (90.0)				
Rural	166	22 (13.3)	144 (86.7)	0.207			
Education background							
Less than high school	170	23 (13.5)	147 (86.5)				
Junior college	402	42 (10.4)	360 (89.6)				
Bachelor's degree	630	64 (10.2)	566 (89.8)				
Master's degree or higher	60	3 (5.0)	57 (95.0)	0.298			
Occupation							
Unemployment	224	23 (10.3)	201 (89.7)				
Manual work	130	21 (16.2)	109 (83.8)				
Non-manual work	908	88 (9.7)	820 (90.3)	0.079			

Samples with CMV loads <3.00 log10 copies/ml were considered negative and considered as samples with no CMV shedding.

VS, vaginal secretion.

*Continuity correction.

**Fig. 2.**
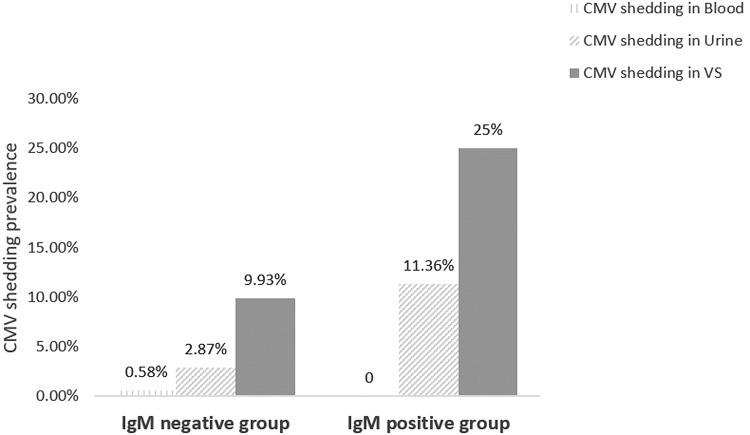
CMV shedding prevalence in blood, urine and vaginal secretion, respectively, in IgM-negative and IgM-positive groups.

**Fig. 3.**
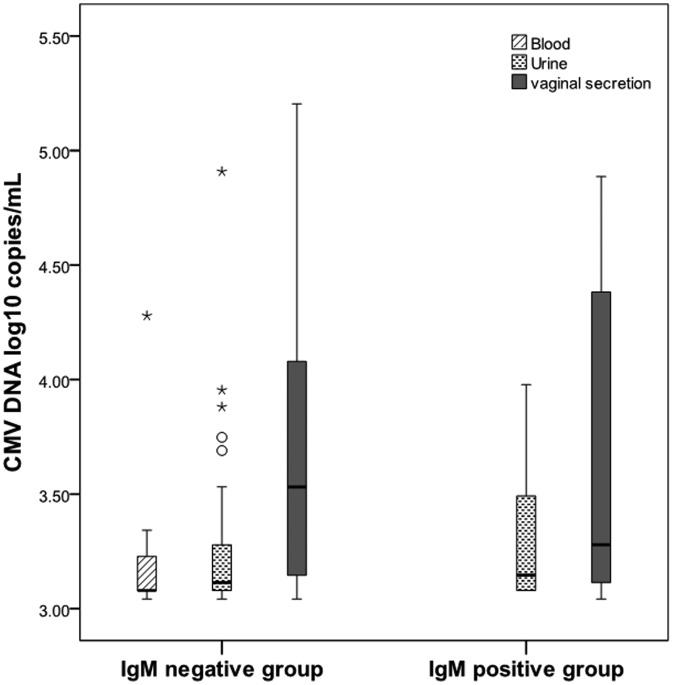
CMV DNA loads (log10 copies/ml) in blood, urine and vaginal secretion samples with positive PCR results (viral loads >3.00 log10 copies/ml) in IgM-negative and IgM-positive groups.

Among the 44 IgM-positive women, 29.5% (13/44) women shed CMV in at least one body fluid, while 12.2% (148/1218) IgM-negative women shed CMV in at least one sample. CMV shedding was more likely detected in IgM-positive women than IgM-negative women (29.5% (13/44) *vs.* 12.2% (148/1218); OR 3.03, 95% CI 1.55–5.93; *P* = 0.001). In the IgM-positive group, 25% (11/44) shed CMV in vaginal secretion and 11.36% (5/44) shed CMV in the urine. There was no one shedding CMV in blood. In the IgM-negative group, 9.93% (121/1218) shed CMV in vaginal secretion, 2.87% (35/1218) shed CMV in urine and 0.58% (7/1218) shed CMV in blood, which were shown in Figure 2. Viral loads in vaginal secretion between IgM-positive and IgM-negative groups were not statistically different (*P* = 0.895). The difference of viral loads in urine between the two groups was also not statistically significant (*P* = 0.751), which was shown in Figure 3.

## Discussion

In our study, we found that among the seropositive women who planned to have babies, 12.8% shed CMV in at least one body fluid. The prevalence of CMV shedding in vaginal secretion was 10.5% which was statistically more frequent than in urine (3.2%) and blood (0.6%), also with higher viral loads. Furthermore, the present study suggested that shedding in vaginal secretion was highly correlated with shedding in urine and the immune state of IgM. In addition, the adverse pregnant history and younger age also affected the prevalence of CMV shedding. These findings may enrich the database on CMV shedding in seropositive immunocompetent women of reproductive age and provide a basis for pregestational health care. It also can provide a clue for further studies.

The shedding prevalence of CMV was well studied among various populations, which always focused on children, pregnancy and immunocompromised people [16–18]. There were few large-scale investigations about the shedding pattern among seropositive women of reproductive age before conception. While several earlier studies investigated the CMV shedding prevalence in non-pregnant women, the sample sizes were small, the detection methods were culture-based and lack sensitivity [22 23]. Meanwhile, the study population was restricted to special patients in STD clinics, infertile couples, postpartum women or even immunocompromised ones [24–26]. A recent study on the prevalence of CMV shedding in healthy seropositive female college students showed that 29.4% (30/102) of the students shed CMV intermittently in saliva or urine. However, the study population was small and the prevalence of CMV shedding in vaginal secretions was not studied [27]. It was documented that the prevalence of CMV shedding was 7.2–33.7% in vaginal secretion and 2.6–10.8% in urine [22, 25–29]. By contrast, the prevalence of CMV shedding in our study was at the lower end. On one hand, the study population in our study was healthy seropositive women, which can represent a normal population of reproductive age. On the other hand, severe immunodeficiency diseases were excluded, which indicated that this population may have better immune control of CMV.

The incidence of CMV DNAemia was just 0.6% among the seropositive women, which was as low as the previously reported findings [29, 30]. Most (6/7) of the women with viremia detected were under low virus loads (range from 3.04 to 3.34 log_10_ copies/ml). Only one woman was detected with a high virus load of 4.28 log_10_ copies/ml in blood without shedding in other body fluids. The low detection rate of viremia could be explained by the transient viremia due to CMV-specific immunity [19]. Consistent with the previous studies, CMV shedding was more frequent in genital secretion than in urine or blood and with higher virus loads. Although we found that shedding in vaginal secretion and urine was highly correlated, the reason why virus shedding differs in one body fluid from another is still unknown. High shedding prevalence and high virus loads in vaginal secretions may hint that the cells in the genital tract are more susceptible to CMV and releasing plenty of CMV DNA off and on as a CMV reservoir [31].

Researches indicated that the CMV seroprevalence was correlated with some sociodemographic factors just like race, region or education level [32]. But CMV shedding prevalence seemed to have no significant association with these factors presented in this study in line with the previous studies. CMV shedding tended to be associated with younger ages, STD, living with young children, lack of running water and cervical inflammation [13, 19, 33]. CMV shedding was more common in children and decreased by age. Although the age span in our study is small, we found that CMV shedding in vaginal secretion was more frequent in women younger than 24, which might be explained by more active sexual act with younger age. We also found that CMV shedding was more likely detected in IgM-positive women than IgM-negative ones, which illustrated that IgM antibody can be taken for a contributory factor in predicting CMV shedding or virus replication. Nevertheless, the viral loads in the two groups made no difference and the positive predictive value of IgM for CMV shedding was just 29% owing to the short persistence of IgM and the low sensitivity and specificity of IgM testing assays [34]. No CMV DNAemia was detected in IgM-positive women, for one thing, the sample size of IgM-positive women was small, for another, there might be a time difference between the formation or disappearance of IgM antibody and the virus entering into blood circulation. As shown above, IgM might be a poor indicator.

Plenty of studies have disclosed that CMV shedding in the first trimester of pregnancy is associated with adverse pregnancy outcomes [14, 15, 35, 36]. Those in the early stage of pregnancy detected CMV DNA-positive in vaginal secretion, especially with high virus loads, may be more prone to miscarriage. In our study, the multiple-factor analysis revealed that adverse pregnancy history was a risk factor for CMV shedding in vaginal secretion. Huang *et al*. suggested that physiologically low loads of CMV shedding in saliva and urine were common and intermittent in healthy seropositive female college students [27]. However, we found that 29.5% (39/132) of women shedding in vaginal secretion were with higher virus loads (>4.0 log_10_ copies/ml). Now for these women, if no intervening measures are taken before gestation, will the intermittent appearance of the virus in the genitourinary tract lead to detrimental results when getting pregnant? Further studies are needed to figure out whether the shedding is occasional or continuous and whether it is associated with adverse pregnancy outcomes.

There were several limitations to our study. Women who were CMV IgG-negative and IgM-positive were not included in the study and we did not test the IgG avidity index. Thus, we could not estimate the relationship between virus shedding and the primary infection. In our current study, the detection method of CMV antibody is qualitative, so we did not research the relationship between anti-CMV IgG level and viral shedding. In addition, the quantification of CMV in vaginal secretion in the comparation was not an absolute quantification owing to the difficulty in precisely controlling the volume of vaginal secretion in sampling and which should be considered as a relative quantification for reference. Another limitation of our study was that we also did not monitor the kinetics of CMV shedding and the variation of the antibody, so there is no evidence to assess the temporal pattern and natural dynamics of CMV shedding. The progression and duration of CMV shedding before gestation and its relationship with adverse pregnancy outcomes should be further investigated.

## Conclusion

CMV shedding was more commonly detected in vaginal secretion than in urine or blood with higher viral loads among healthy seropositive women of reproductive age. The presence of IgM, shedding in urine, younger age and adverse pregnancy history were associated with the virus shedding in vaginal secretion. More attention should be paid to those who have been detected CMV DNA-positive in vaginal secretion or other body fluids before the intended pregnancy, especially with adverse pregnancy history. Further studies are needed to figure out whether the shedding is occasional or continuous and whether it is associated with adverse pregnancy outcomes.
